# Effectiveness of decision support tools on reducing antibiotic use for respiratory tract infections: a systematic review and meta-analysis

**DOI:** 10.3389/fphar.2023.1253520

**Published:** 2023-09-07

**Authors:** Rixiang Xu, Lang Wu, Lingyun Wu, Caiming Xu, Tingyu Mu

**Affiliations:** ^1^ School of Humanities and Management, Zhejiang Chinese Medical University, Hangzhou, Zhejiang, China; ^2^ School of Nursing, Zhejiang Chinese Medical University, Hangzhou, Zhejiang, China; ^3^ School of Law, Hangzhou City University, Hangzhou, Zhejiang, China; ^4^ School of Nursing, Anhui Medical University, Hefei, Anhui, China

**Keywords:** clinical decision support, antimicrobials, substance abuse, respiratory tract infections, antimicrobial stewardships

## Abstract

**Background:** Clinical decision support tools (CDSs) have been demonstrated to enhance the accuracy of antibiotic prescribing among physicians. However, their effectiveness in reducing inappropriate antibiotic use for respiratory tract infections (RTI) is controversial.

**Methods:** A literature search in 3 international databases (Medline, Web of science and Embase) was conducted before 31 May 2023. Relative risk (RR) and corresponding 95% confidence intervals (CI) were pooled to evaluate the effectiveness of intervention. Summary effect sizes were calculated using a random-effects model due to the expected heterogeneity (*I* ^*2*^ over 50%).

**Results:** A total of 11 cluster randomized clinical trials (RCTs) and 5 before-after studies were included in this meta-analysis, involving 900,804 patients met full inclusion criteria. Among these studies, 11 reported positive effects, 1 reported negative results, and 4 reported non-significant findings. Overall, the pooled effect size revealed that CDSs significantly reduced antibiotic use for RTIs (RR = 0.90, 95% CI = 0.85 to 0.95, *I* ^*2*^ = 96.10%). Subgroup analysis indicated that the intervention duration may serve as a potential source of heterogeneity. Studies with interventions duration more than 2 years were found to have non-significant effects (RR = 1.00, 95% CI = 0.96 to 1.04, *I* ^*2*^ = 0.00%). Egger’s test results indicated no evidence of potential publication bias (*p* = 0.287).

**Conclusion:** This study suggests that CDSs effectively reduce inappropriate antibiotic use for RTIs among physicians. However, subgroup analysis revealed that interventions lasting more than 2 years did not yield significant effects. These findings highlight the importance of considering intervention duration when implementing CDSs.

**Systematic Review Registration:** https://www.crd.york.ac.uk/prospero/display_record.php?ID=CRD42023432584, Identifier: PROSPERO (CRD42023432584).

## 1 Introduction

Respiratory tract infections (RTIs) commonly encountered in medical practice include otitis media, sinusitis, pharyngitis, acute bronchitis, and pneumonia (Gulliford et al., 2014a). These acute respiratory infections are predominantly viral in origin and tend to be self-limiting (Peters et al., 2011; Wang et al., 2021). Despite their viral etiology, antibiotics are commonly prescribed in clinical practice for their treatment. Inappropriate antibiotic use has been observed in 50%–88% of cases involving RTI patients, highlighting a pervasive trend of antibiotics overprescription (Botica et al., 2013; Shen et al., 2021; Calabria et al., 2022). This pattern serves as a classic manifestation of antibiotic misuse, which not only fails to effectively alleviate respiratory symptoms but also contributes to the emergence and dissemination of antimicrobial resistance. Moreover, the lack of microbiological testing and indiscriminate use of broad-spectrum antibiotics in the management of bacterial RTIs further exacerbate the development of antibiotic resistance (Webb et al., 2019a; Zhang et al., 2022). Studies indicate that globally, at least 4.95 million people deaths associated with antibiotic-resistant infections each year, and it is expected to lead to a global economic output loss of about 100 trillion USD by 2050 (O’Neill, 2014; Antimicrobial Resistance Collaborators, 2022). These alarming circumstances have prompted the World Health Organization (WHO) to acknowledge bacterial resistance as a major global health concern, urging nations worldwide to implement stringent measures for the regulation of antimicrobial agents (WHO, 2021). In line with these concerns, current treatment guidelines emphasize a no antibiotic prescribing strategy for the majority of self-limiting RTIs, as evidence suggests that reducing antibiotic prescribing does not compromise patient safety regarding bacterial infections (Gerber et al., 2017; Webb et al., 2019a). This strategy is crucial for curtailing antibiotic overuse, mitigating the rise of antimicrobial resistance, and safeguarding public health.

**FIGURE 1:**
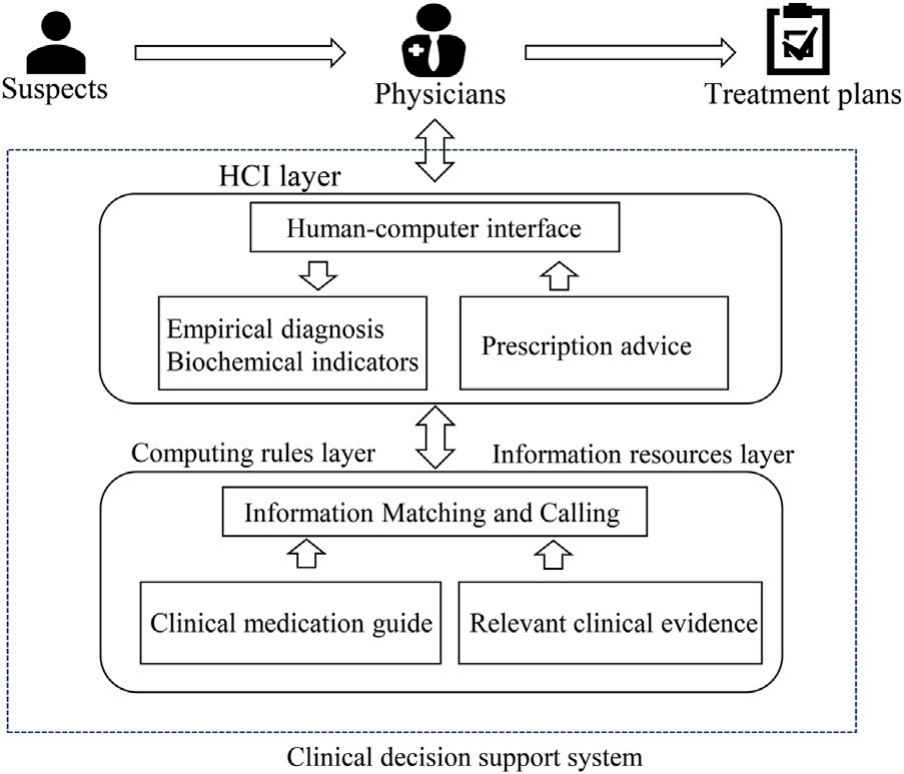
Operational model of a clinical decision support system for antibiotic prescribing (HCI, human-computer interaction).

To address the challenges associated with antibiotic prescribing in RTIs, clinical decision support tools (CDSs) have emerged as invaluable computer programs that aid physicians in making evidence-based treatment decisions (Eudaley et al., 2019). These tools incorporate guidelines and relevant clinical evidence into their algorithms to provide tailored decision support based on patient diagnostic outcomes (New et al., 2022). Typically integrated within hospital information systems and seamlessly connected to electronic health records, CDSs hold great potential for facilitating accurate prescribing practices in clinical settings, thereby mitigating the incidence of antibiotic misuse resulting from physician errors. However, it is important to note that these tools serve as supportive aids and should not supplant the role of physicians in making prescription decisions (Jones et al., 2023). Figure 1 represents a common flow chart of CDSs for antibiotic prescriptions.

The effectiveness of clinical decision support tools has been a subject of debate in the literature. While some studies have demonstrated their ability to significantly improve prescribing behavior (Gonzales et al., 2013; Gulliford et al., 2019), others have questioned their efficacy or even suggested potential negative impacts on prescribing practices (Mostaghim et al., 2019; van de Maat et al., 2020). Consequently, a systematic review and meta-analysis have been conducted to critically evaluate existing studies and provide comprehensive insights into the application of CDSs in clinical settings, aiming to establish a clear understanding of their overall impact and utility.

## 2 Methods

This systematic review and meta-analysis followed PRISMA guidelines for transparent reporting. The study protocol was registered in PROSPERO (CRD42023432584).

### 2.1 Search strategy

A comprehensive literature search was conducted in three databases: PubMed, Web of Science, and Embase. The search was conducted up until 31 May 2023, and included the following search terms: “Decision Support Systems, Clinical,” “Anti-Bacterial Agents,” “Acute Respiratory Infections,” and “Respiratory Tract Infections.” The search strategy aimed to identify relevant studies that examined the impact of decision support tools on reducing antibiotic use in the context of acute respiratory infections.

### 2.2 Study selection

Two independent reviewers screened the titles and abstracts of the identified articles to determine their eligibility for inclusion. In cases of disagreement, a third reviewer was consulted. Full-text articles of potentially eligible studies were retrieved and further assessed for inclusion based on predefined criteria. The study selection process followed the PICOS (Population, Intervention, Comparison, Outcome, Study design) framework. Participants (P) included patients diagnosed with acute respiratory tract infections. The intervention (I) involved the use of decision support tools by healthcare professionals. The comparison (C) group consisted of those without decision support tools or a pre-intervention comparison. The primary outcomes (O) measured were antibiotic prescribing rates or quantifiable effect sizes with 95% confidence intervals. The study designs (S) considered were randomized clinical trials and observational studies published in English.

### 2.3 Data extraction

Data extraction was performed independently by two reviewers using a standardized data extraction form. The extracted information included study characteristics (e.g., author, year of publication, study design), participant characteristics (e.g., sample size, demographics), details of the CDSs used (e.g., type, features), antibiotic prescribing outcomes (e.g., as prescription rate or RR or OR value and corresponding 95% CI), and the conclusion of statistical analysis.

### 2.4 Risk of bias assessment

In this systematic review and meta-analysis, the methodological quality of the included studies was assessed using specific tools to evaluate bias. The RoB 2.0 tool assessed randomized clinical trials (RCTs) across six key domains: Randomization, Interventions, Missing outcome data, Outcome Measurement, Reported results, and other sources of bias. Each domain was assigned a risk level: low, uncertain, or high. This comprehensive assessment provided insights into RCT quality and potential biases (Sterne et al., 2019). For before-and-after studies, the NIH Quality Assessment Tool focused on 12 domains, including study design, blinding, allocation concealment, participant selection, data collection methods, and statistical analysis, etc. Each domain was categorized as good, fair, or poor, indicating quality and potential biases (NIH, 2013). The risk of bias assessment was conducted independently by three authors and resolved through discussions or review by a fourth author. This rigorous evaluation using standardized tools enhanced the reliability and validity of the findings. Publication bias was assessed using funnel plots and statistical tests, such as Egger’s regression test, to detect potential publication bias or small-study effects.

### 2.5 Standardization of data

In order to enhance comparability among studies with different types of outcome measures, standardization of data was performed in this study. Prescription rates, prescription change rates, and odds ratios (ORs) were transformed into RRs for consistent analysis. Firstly, prescription change rates were divided by 100 and added by 1 to obtain the RR value. The transformation of prescription rates and ORs into RRs was conducted according to Eq. 1, 2 respectively (Viera, 2008; Wang et al., 2018).

$$RR=P_{1}/P_{0}$$ (1)

$$RR=OR/\left[\left(1-P_{0}\right)+\left(P_{0}\times OR\right)\right]$$ (2)

Where *P*_*1*_ and *P*_*0*_ is prescription rates with intervention or without intervention independently.

This standardized approach allowed for a more uniform comparison and interpretation of the results across different outcome measures.

### 2.6 Statistical analysis

Meta-analysis was performed using standardized RRs and their corresponding 95% CIs extracted or re -calculate from each eligible study. The presence of heterogeneity among the included studies was evaluated utilizing Cochran’s *Q* test and the *I* ^*2*^ statistic. A significance level of *p* < 0.1 for Cochran’s *Q* test was considered indicative of statistically significant heterogeneity. The *I* ^*2*^ statistic provided a quantitative measure of heterogeneity and inconsistency across studies. Consistent with prior research, *I* ^*2*^ values of 0%–25%, 25%–50%, 50%–75%, and 75%–100% were interpreted as representing no heterogeneity, mild heterogeneity, moderate heterogeneity, and substantial heterogeneity, respectively (Xu et al., 2018).

Given the observed substantial heterogeneity in this study, a random-effects model was selected to pool the effect sizes. Subsequently, a subgroup analysis was conducted to explore potential sources of heterogeneity. To assess publication bias, visual inspection of funnel plots for asymmetry and Egger’s test were employed, with a significance level of *p* < 0.05 indicating the presence of publication bias. The meta-analyses were conducted using STATA version 13.0 (Stata Corporation, TX, United States), ensuring robust and reliable data synthesis.

## 3 Results

### 3.1 Literature retrieval and study characteristics

**FIGURE 2:**
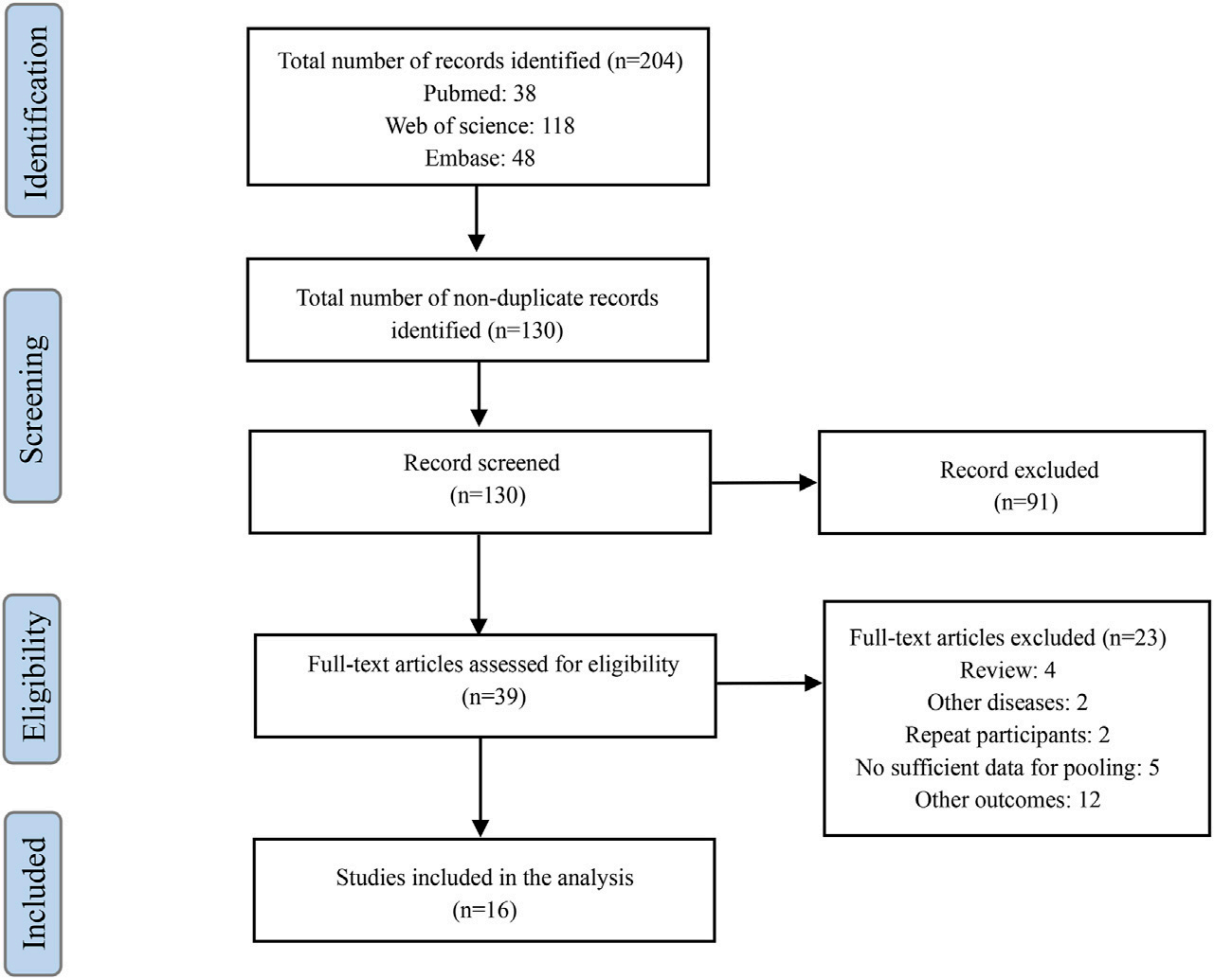
Flow chart of the study selection process.

A total of 204 potential articles were identified through our search strategy. After reviewing titles and abstracts, 130 articles were retained after removing duplicates and irrelevant studies. Further evaluation of the full texts based on our inclusion and exclusion criteria resulted in the exclusion of 91 articles. Ultimately, 16 relevant studies were included in our analysis (Figure 2) (Samore et al., 2005; Linder et al., 2009; Bourgeois et al., 2010; Gonzales et al., 2013; Litvin et al., 2013; Mcginn et al., 2013; Gulliford et al., 2014b; Meeker et al., 2016; Vervloet et al., 2016; Sharp et al., 2017; Webb et al., 2019b; Gulliford et al., 2019; Mostaghim et al., 2019; Devin Mann et al., 2020; van de Maat et al., 2020; May et al., 2021).

The characteristics of the included studies are presented in Table 1, involving a total of 900,804 participants. Among them, 5 studies had a before-after study design, while 11 studies were randomized clinical trials (including two studies with two intervention comparisons). The duration of the studies ranged from 6 to 33 months. Geographically, the studies were conducted in developed countries such as the United States, Germany, Sweden, and Australia. Participants were diagnosed with respiratory tract infections, including acute bronchitis (1 study), acute sinusitis (1 study), community-acquired pneumonia (2 studies), and acute respiratory tract infections (9 studies). Most interventions utilized computerized CDS, while two studies employed printed CDS. In terms of outcomes, 11 studies reported statistically significant reductions in antibiotic prescription rates with the use of CDS, 4 studies reported non-significant findings, and 1 study reported an increase in antibiotic utilization associated with CDS.

**TABLE 1:** Characteristics of studies included in the analysis.

Name (publication year)	Country	Study design	Duration	Population size	Diagnosis	Interventions	Outcome [RR (95% CI)]	Conclusion
Gonzales et al. (2013)	American	RCT	6 months	Group 1: pre- = 3,639, post- = 1,001	Acute Bronchitis	Group 1: Printed decision support	Group 1: 0.869 (0.796–0.958)	Significantly reduced
				Group 2: pre- = 2,974, post- = 1,017		Group 2: computer-assisted decision support intervention	Group 2: 0.872 (0.759–0.975)	
				Control group: pre- = 3,195, post- = 950				
Litvin et al. (2013)	American	BAS	27 months	Adults: 13,285	ARIs	Computer-assisted decision support intervention	Adult: 1.016 (0.9465–1.0849)	No statistically different
				Children: 6,467			Children: 0.9811 (0.9097–1.0526)	
Gulliford et al. (2019)	United Kingdom	RCT	12 months	Intervention group: 323 155	RTI	Computer-assisted decision support intervention	0.88 (0.78–0.99)	Significantly reduced
				Control group: 259 520				
Gulliford et al. (2014a)	United Kingdom	RCT	12 months	Intervention group: pre- = 59,226, post- = 59,309	RTI	Computer-assisted decision support intervention	0.987 (0.975–0.999)	Significantly reduced
				Control group: pre- = 54,431, post- = 51,093				
Linder et al. (2009)	American	RCT	7 months	Intervention group: 594	ARI	Computer-assisted decision support intervention	1.142 (1.066–1.223)	Significantly increased
				Control group: 6,236				
McGinn et al. (2013)	American	RCT	12 months	Intervention group: 586	ARI	Computer-assisted decision support intervention	0.74 (0.60–0.92)	Significantly reduced
				Control group: 398				
Devin Mann et al. (2020)	American	RCT	33 months	Intervention group: 42,126	ARI	Computer-assisted decision support intervention	0.99 (0.87–1.11)	No statistically different
				Control group: 58,447				
May et al. (2021)	American	BAS	6 months	Pre-intervention: 306	ARI	Computer-assisted decision support intervention	0.846 (0.767–0.932)	Significantly reduced
				Post-intervention: 263				
Mostaghim et al. (2018)	Australia	BAS	24 months	Pre-intervention: 134	Pneumonia	Computer-assisted decision support intervention	1.005 (0.911–1.108)	No statistically different
				Post-intervention: 107				
Webb et al. (2019a)	Australia	BAS	12 months	Pre-intervention: 1,122	Pneumonia	Computer-assisted decision support intervention	0.825 (0.738–0.923)	Significantly reduced
				Post-intervention: 1,047				
Sharp et al. (2017)	American	BAS	6 months	Pre-intervention: 10,491	Acute sinusitis	Computer-assisted decision support intervention	0.962 (0.946-0.979)	Significantly reduced
				Post-intervention: 11,458				
van de Maat et al. (2020)	Netherlands	RCT	33 months	Pre-intervention: 597	lower RTI	Computer-assisted decision support intervention	1.05 (0.65–1.54)	No statistically different
				Post-intervention: 402				
Meeker et al. (2016)	American	RCT	18 months	Intervention group: 2,388	ARI	Computer-assisted decision support intervention	0.467 (0.386–0.566)	Significantly reduced
				Control group: 2,095				
Vervloet et al. (2016)	Netherlands	RCT	12 months	Intervention group: 59,483	RTI	Computer-assisted decision support intervention	0.775 (0.757–0.794)	Significantly reduced
				Control group: 94,767				
Bourgeois et al. (2010)	American	RCT	6 months	Intervention group: 419	ARI	Computer-assisted decision support intervention	0.795 (0.690–0.916)	Significantly reduced
				Control group: 14,515				
Samore et al. (2005)	American	RCT	9 months	Adults: 5,886	ARI	Printed and Computer-assisted decision support intervention	0.893 (0.843–0.949)	Significantly reduced
				Children: 7,195				

ARI, acute respiratory infections; RTI, respiratory tract infections; RR, relative risk; CI, confidence interval; RCT, randomized clinical trial; BAS, before-after study.

### 3.2 Meta-analysis results

**FIGURE 3:**
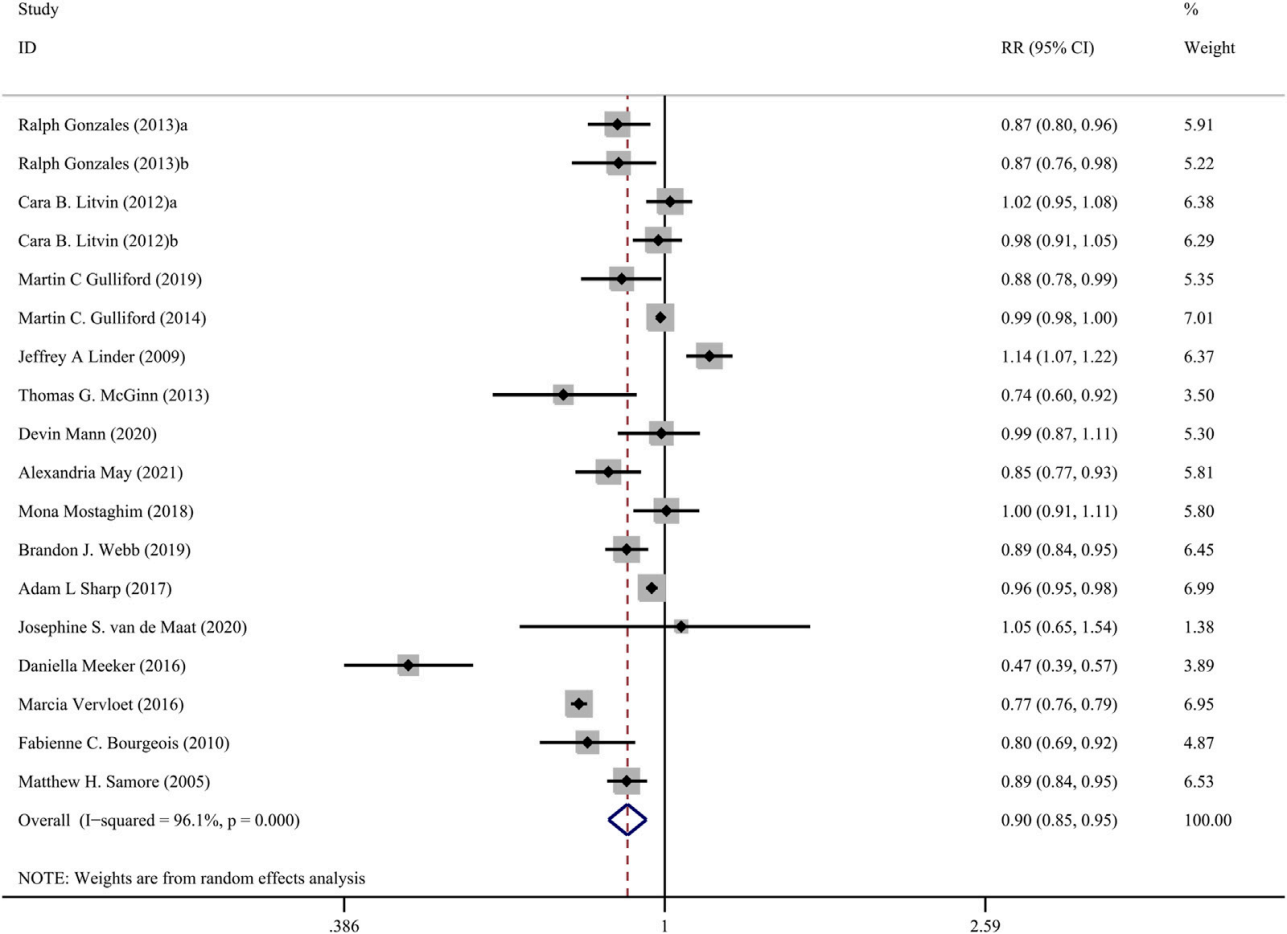
Forest plot of the effect of CDSs on reducing antibiotic use for RTIs.

**FIGURE 4:**
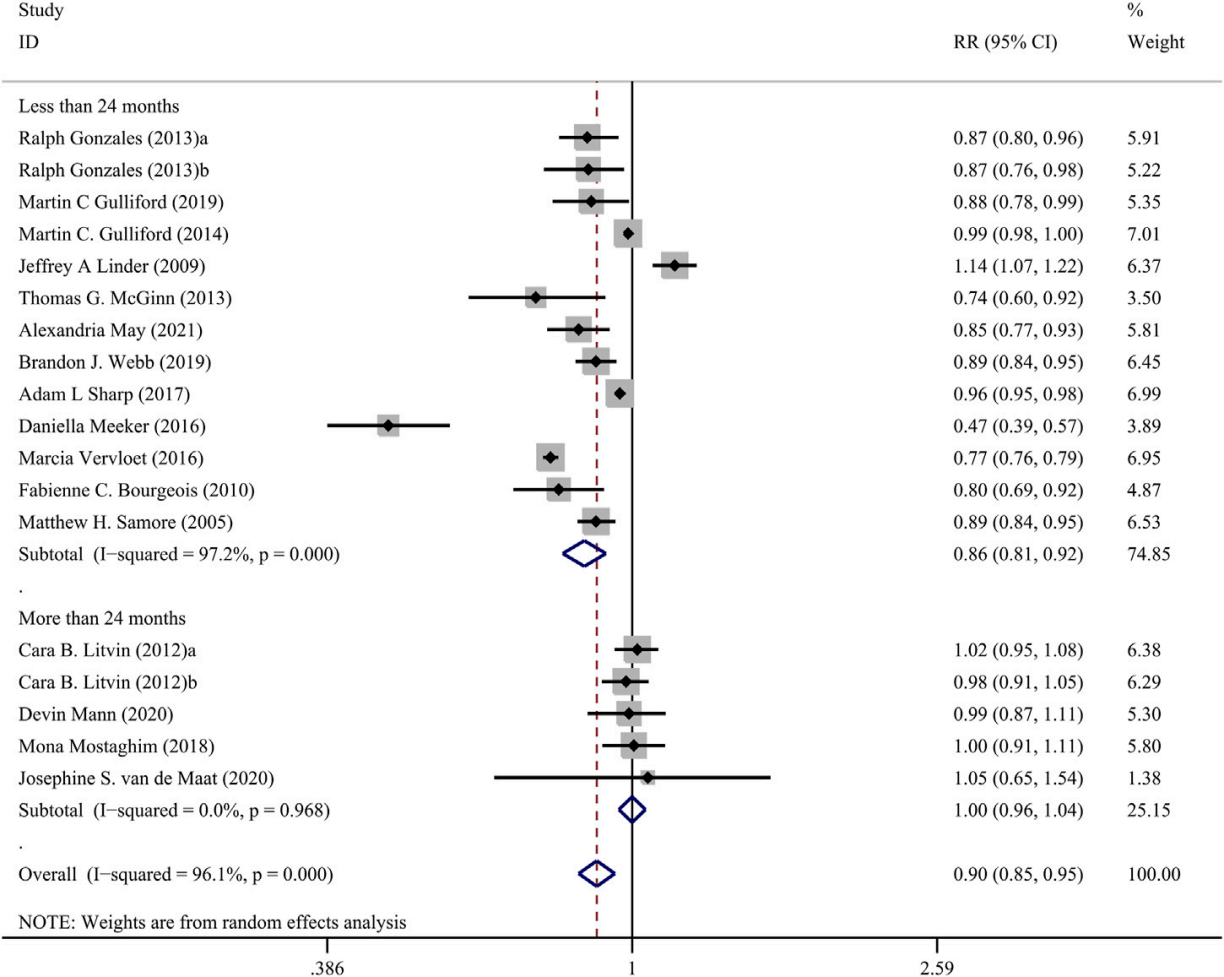
Forest plot of subgroup analysis of intervention duration of CDSs on reducing antibiotic use for RTIs.

The Figure 3 presents the pooled results of 16 studies on CDS interventions to reduce antibiotic prescribing rates in patients with respiratory tract infections. Due to substantial heterogeneity, a random-effects model was chosen. The results indicate a moderate effect of CDS in reducing antibiotic prescribing rates among patients with respiratory tract infections (RR = 0.90, 95% CI = 0.85–0.95, *I* ^*2*^ = 96.10%). Subgroup analysis revealed that interventions with a duration of 24 months or longer did not show significant effects (RR = 1.00, 95% CI = 0.96–1.04, *I* ^*2*^ = 0.00%), while interventions with a duration of less than 24 months significantly reduced antibiotic prescribing rates (RR = 0.86, 95% CI = 0.81–0.92, *I* ^*2*^ = 97.20%) (Figure 4).

### 3.3 Risk of bias assessment

**FIGURE 5:**
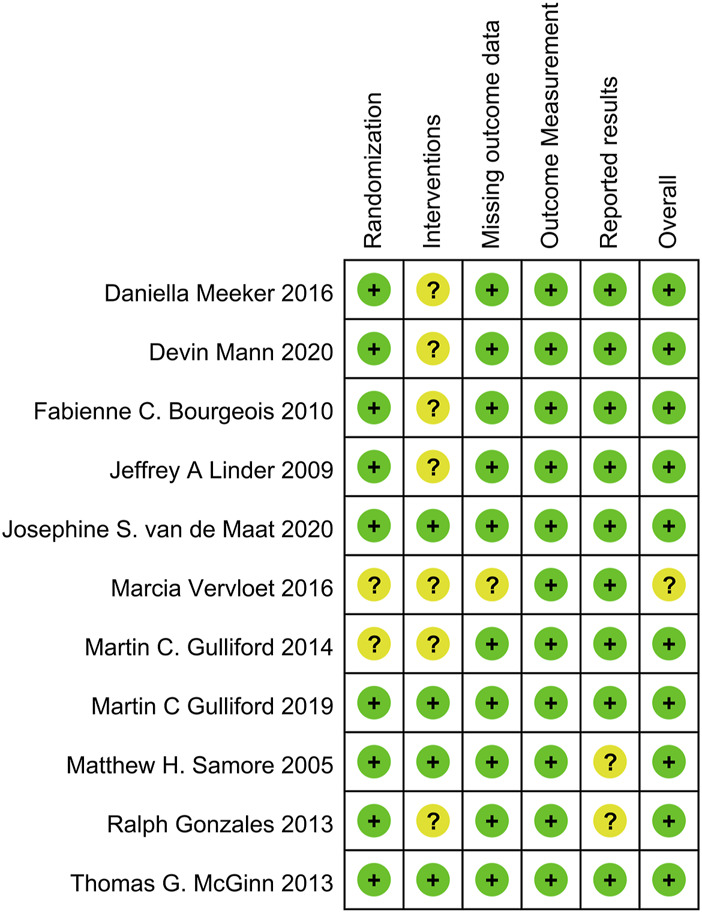
Risk assessment of bias for RCTs using ROB 2.0 tool.

**FIGURE 6:**
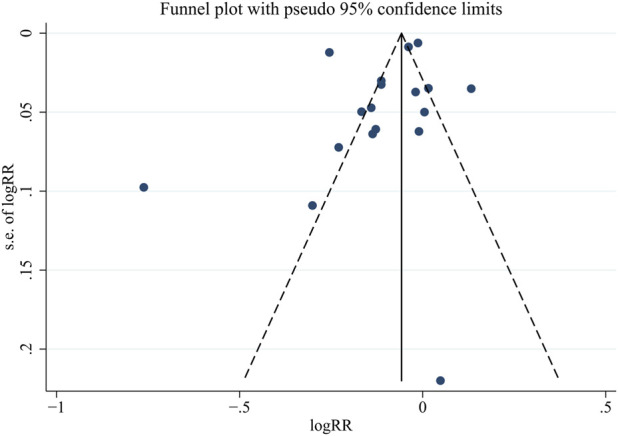
Funnel plot to evaluate potential publication bias.

Supplementary Table S1 and Figure 5 present the risk of bias assessment for before-after studies and RCTs, respectively. Among the 5 before-after studies, 3 were rated as good (low risk) and 2 as fair (medium risk). Among the 11 RCTs, 10 were considered low risk, and 1 had an uncertain risk. The funnel plot for publication bias assessment (Figure 6) visually demonstrates symmetry. The results of Egger’s test suggest a low risk of publication bias (*p* = 0.287).

## 4 Discussion

Our study aimed to evaluate the effectiveness of CDSs in reducing antibiotic prescription rates for RTIs through a systematic review and meta-analysis. The results of our study revealed a modest effect of decision support tools in reducing antibiotic use, indicating that their implementation can promote more judicious prescribing practices in RTI management. However, it is important to consider factors that may influence healthcare providers’ adherence to the recommendations provided by CDSs.

One possible explanation for the observed modest effect is partial adherence by physicians to the recommendations provided by CDSs. Despite the availability of evidence-based guidelines and CDSs, healthcare providers may deviate from these recommendations for various reasons. Firstly, physicians may not fully adhere to the recommendations provided by the CDSs due to concerns about potential adverse outcomes. It is possible that the fear of missing a bacterial infection or the fear of adverse events may influence physicians’ decision-making processes, leading to a cautious approach to antibiotic prescribing (Ashiru-Oredope et al., 2021; Mmari et al., 2021; Xu et al., 2021). Secondly, patient preferences and demands may also contribute to the observed effect. Patients may insist on receiving antibiotics despite recommendations from CDSs, which can create challenges for physicians in adhering to evidence-based practices (Finkelstein et al., 2014; Duan et al., 2022). Additionally, financial incentives or other external factors may influence prescribing behaviors, leading to deviations from the recommendations provided by CDSs (Liu et al., 2019). Identifying these challenges and addressing them through targeted interventions and educational initiatives can improve the utilization and implementation of CDSs.

Comparing our findings with previous research on the use of CDSs in other clinical settings provides valuable insights. Similar to our study, the meta-analysis on CDSs and antibiotic prescribing found that decision support systems improve the precision of clinical antibiotic prescriptions and reduce prescription rates in primary care settings (Carracedo-Martinez et al., 2019). However, there was no observed improvement in mortality rates and hospital stay (Carracedo-Martinez et al., 2019). Nevertheless, previous systematic reviews demonstrated that CDSs can enhance prescribing accuracy and reduce adverse events among physicians (Reis et al., 2017; Whitehead et al., 2019). However, a meta-regression analysis suggested that CDS integrated with electronic chart or order entry systems is unlikely to effectively improve the process of care or patient outcomes (Roshanov et al., 2013). These variations may stem from differences in study populations, healthcare systems, implementation strategies, and the complexity of clinical decision-making. These variations contribute to the understanding of the varied impacts of CDCs in different healthcare contexts.

Subgroup analysis revealed that the duration of the intervention may serve as a potential source of heterogeneity. Studies with interventions lasting more than 2 years showed non-significant effects on antibiotic prescribing rates (RR = 1.00, 95% CI = 0.96–1.04). This suggests that the sustained use of decision-support tools over an extended period may lead to a reduction in their effectiveness. Longer intervention durations could amplify the influence of the aforementioned factors (concerns, patient insistence, and extrinsic motivations) on physicians or a loss of enthusiasm for the CDSs over time. Future studies should explore strategies to ensure the long-term sustainability and effectiveness of decision-support interventions in clinical practice.

The practical implications of our research findings are noteworthy. The implementation of CDSs holds the potential to optimize antibiotic prescribing practices and address the growing problem of antimicrobial resistance. By providing evidence-based recommendations to clinicians in clinical practice, CDSs have the potential to improve clinical decision-making, enhance patient outcomes, and reduce unnecessary antibiotic use. However, to maximize their impact, comprehensive measures are needed, including integrating CDSs into electronic health records, providing ongoing education and training for healthcare providers, and fostering a culture of antimicrobial resistance management.

Despite the overall positive effects observed, it is crucial to acknowledge the limitations of this study. There are several limitations to this study. Firstly, the majority of included studies were conducted in developed countries such as Europe, the United States, and Australia. The lack of research from underdeveloped countries may limit the generalizability of the findings. Secondly, some studies were conducted over a decade ago. Given the rapid advancement of information technology, newer CDSs may offer improved user experiences to physicians, potentially leading to better outcomes in antibiotic prescribing. Lastly, substantial heterogeneity was observed in the analysis results, caution is advised when interpreting and applying the findings of this study.

## 5 Conclusion

In conclusion, our study contributes to the growing body of evidence supporting the effectiveness of CDSs in reducing antibiotic prescription rates for RTIs. Despite the observed modest effect, barriers to adherence and contextual factors need to be addressed to ensure optimal utilization of these tools. Future research should focus on strategies to enhance the implementation of CDSs, assess their long-term sustainability, and evaluate their impact on patient outcomes and antimicrobial resistance. Ultimately, successful integration of CDSs into clinical practice holds the potential to transform antibiotic prescribing practices and improve public health outcomes.

## Data Availability

The original contributions presented in the study are included in the article/Supplementary Material, further inquiries can be directed to the corresponding authors.
