# Supplementary material for: Effectiveness of decision support tools on reducing antibiotic use for respiratory tract infections: a systematic review and meta-analysis
Source: Front Pharmacol. 2023 Sep 7;14:1253520. doi: 10.3389/fphar.2023.1253520 (PMC10512864; doi:10.3389/fphar.2023.1253520)
Supplement: Supplementary file 1 [file Table1.DOCX]

Supplementary Material

# Supplementary Table

| Study | 1 | 2 | 3 | 4 | 5 | 6 | 7 | 8 | 9 | 10 | 11 | 12 | Overall |
| --- | --- | --- | --- | --- | --- | --- | --- | --- | --- | --- | --- | --- | --- |
| Cara B. Litvin 2012 | Y | NR | Y | Y | Y | Y | Y | N | Y | Y | NA | Y | Good |
| Alexandria May 2021 | Y | Y | Y | Y | Y | Y | Y | N | Y | Y | NA | N | Fair |
| Mona Mostaghim 2018 | Y | Y | Y | Y | N | Y | Y | N | Y | Y | NA | N | Fair |
| Brandon J. Webb 2019 | Y | NR | Y | Y | Y | Y | Y | N | Y | Y | Y | N | Good |
| Adam L Sharp 2017 | Y | Y | Y | Y | Y | Y | Y | N | Y | Y | Y | Y | Good |
| Abbreviations: Y, yes; N, no; NA, not applicable; NR, not reported. 1. objective clearly stated; 2. eligibility criteria described; 3. representative patient population; 4. all eligible participants enrolled in study; 5. sufficient sample size; 6. intervention described; 7. outcome measures specified; 8. outcome assessors blinded; 9. loss to follow-up; 10. statistical analysis of outcome measures before and after intervention; 11. interrupted time-series design; 12. individual data used for group-level effects. | | | | | | | | | | | | | |

Supplementary table 1. Risk assessment of bias for before-after studies.
